# Amplifying the Heat Shock Response Ameliorates ALS and FTD Pathology in Mouse and Human Models

**DOI:** 10.1007/s12035-023-03509-2

**Published:** 2023-07-29

**Authors:** Mhoriam Ahmed, Charlotte Spicer, Jasmine Harley, J. Paul Taylor, Michael Hanna, Rickie Patani, Linda Greensmith

**Affiliations:** 1 Department of Neuromuscular Diseases, UCL Queen Square Institute of Neurology, London, WC1N 3BG UK; 2 The Francis Crick Institute, 1 Midland Road, London, NW1 1AT UK; 3 Department of Cell and Molecular Biology, St. Jude Children’s Research Hospital, Memphis, USA

**Keywords:** ALS, FTD, Dementia, VCP, Heat shock response, Proteostasis, Motor neuron, Therapy, Treatment

## Abstract

Amyotrophic lateral sclerosis (ALS) and frontotemporal dementia (FTD) are now known as parts of a disease spectrum with common pathological features and genetic causes. However, as both conditions are clinically heterogeneous, patient groups may be phenotypically similar but pathogenically and genetically variable. Despite numerous clinical trials, there remains no effective therapy for these conditions, which, in part, may be due to challenges of therapy development in a heterogeneous patient population. Disruption to protein homeostasis is a key feature of different forms of ALS and FTD. Targeting the endogenous protein chaperone system, the heat shock response (HSR) may, therefore, be a potential therapeutic approach. We conducted a preclinical study of a known pharmacological amplifier of the HSR, called arimoclomol, in mice with a mutation in valosin-containing protein (VCP) which causes both ALS and FTD in patients. We demonstrate that amplification of the HSR ameliorates the ALS/FTD-like phenotype in the spinal cord and brain of mutant VCP mice and prevents neuronal loss, replicating our earlier findings in the SOD1 mouse model of ALS. Moreover, in human cell models, we demonstrate improvements in pathology upon arimoclomol treatment in mutant VCP patient fibroblasts and iPSC-derived motor neurons. Our findings suggest that targeting of the HSR may have therapeutic potential, not only in non-SOD1 ALS, but also for the treatment of FTD.

**Supplementary Information:**

The online version contains supplementary material available at 10.1007/s12035-023-03509-2.

## Introduction

Although amyotrophic lateral sclerosis (ALS) and frontotemporal dementia (FTD) have been traditionally categorised as distinct neurodegenerative disorders, it is now widely accepted that they form part of a degenerative disease continuum [1], with overlapping genetic, clinical and pathological features. A major genetic link between familial ALS and FTD was identified in 2011, with the discovery of the GGGGCC pathogenic repeat expansions in the *C9orf72* gene, which accounts for approximately 25% of familial FTD and 40% of familial ALS cases [2, 3]. Other, less frequently occurring mutations including those in *TARDBP*, *SQSTM1* and *VCP* genes [4] have also been identified, although the precise mechanism underlying the resulting pathology has yet to be fully determined for any of these mutations.

Mutations in the valosin-containing protein (VCP/p97) gene on chromosome 9p13-p12 are associated with both familial FTD (<1%) and familial ALS (1–2%) [5, 6]. Expression of mutant VCP leads to a multisystem inherited pleiotropic disorder that can affect muscle, bone and the nervous system, now referred to as multisystem proteinopathy (MSP), but previously referred to by the acronym IBMPFD (inclusion body myopathy (IBM) with Paget’s disease of bone (PDB) and FTD [7, 8]. Patients with MSP present with a combination of clinical and pathological symptoms that includes ALS as well as FTD, IBM and PDB. Whilst ALS, FTD and IBM pathology are distinct in each tissue and can exist independently, they share common pathological hallmarks which are indicative of protein dyshomeostasis, with the presence of ubiquitin-positive inclusion bodies, p62 aggregation and mislocalisation of the RNA-binding protein, TDP-43, observed in all three disorders [9–11] .

VCP is a ubiquitously expressed protein which forms a monohexamer containing three main domains: The N domain, mainly associated with substrate binding, the D1 domain for oligomerisation and ATP hydrolysis and the D2 domain, which also undertakes ATPase activity [12]. VCP associates with a large range of co-factors to bring about diverse cellular functions ranging from progression of the cell cycle to membrane fusion [12]. As a segregase, powered by ATP hydrolysis, VCP extracts and unfolds substrate proteins ubiquitinated for degradation. VCP is also linked to maturation of autophagosomes which are crucial for autophagy to take place [13]. This protein is therefore heavily involved in the two major protein degradation mechanisms of the cell: autophagy and the ubiquitin-proteasome system (UPS) [14, 15]. It therefore follows that mutations in VCP may alter cellular protein homeostasis by dysregulating these important housekeeping systems. This in turn is likely to increase the aberrant protein load in a cell leading to proteinaceous aggregate formation, potentially accounting for the degenerative pathology seen in the spinal cord, brain and muscle of MSP patients with mutant VCP and linking protein dyshomeostasis as a pathomechanism in other ALS/FTD cases.

Restoration of protein homeostasis may therefore be a potential therapeutic approach to ameliorate pathology in MSP and ALS/FTD. In this study, we examined the possibility that targeting the endogenous protein handling mechanism known as the heat shock response (HSR) may be a successful strategy to restore protein homeostasis in MSP and ALS/FTD. The HSR is a ubiquitous, cytoprotective signalling pathway that is triggered under conditions of acute or chronic stress, leading to activation of the transcription factor heat shock factor 1 (HSF-1) [16–20] and subsequent upregulation of the family of molecular chaperones known as heat shock proteins (HSPs). HSPs sequester hydrophobic regions of misfolded proteins to prevent protein-protein interaction and subsequent aggregation. HSPs are also involved in regulating lysosomal function and autophagy by delivering aberrant proteins to the surface of lysosomes and enabling autophagic degradation [21]. Indeed HSF-1 itself has been shown to regulate autophagy in the cell, such as by altering SQSTM1/p62 phosphorylation and at the transcriptional level by upregulating *Atg* genes required for autophagy [22, 23]. In addition, HSP70, a major player in the chaperone system, has been shown to stabilise lysosomal integrity [24–26]. Therefore, upregulating the expression of HSPs in protein-misfolding diseases such as MSP and ALS/FTD may be an effective approach to restore the proteostasis impaired by aberrant proteins and improve cellular health.

We have previously tested the potential therapeutic effects of amplifying the HSR using a pharmacological agent called arimoclomol. Treatment with arimoclomol improved both neuropathological and functional deficits in mouse models of two motor neuron diseases—the SOD1^G93A^ mouse model of ALS [27–29] and the AR100 mouse model of spinal and bulbar muscular atrophy (SBMA) [30]. Arimoclomol has been shown to act, at least in part, like a smart-drug, prolonging the activation of HSF-1 by augmenting its DNA-binding potential, but only in cells in which the HSR has already been activated in response to stress [19, 27, 31]. Although the exact molecular mechanism by which arimoclomol, and similar agents such as bimoclomol, BGP-15 or any of their analogues *co-induce* the HSR (i.e. amplify the HSR in cells under stress and already mounting this cytoprotective response) remains unresolved, their unique ability to *amplify* rather than *activate* the HSR makes them particularly attractive therapeutic compounds as they avoid the off-target side effects of widespread, non-specific activation of the HSR in multiple cell types, including those not affected in disease and not under stress; this is a major drawback of other approaches to upregulate the HSR.

We have also shown that amplification of the HSR by treatment with arimoclomol ameliorates skeletal muscle pathology in models of IBM, including in mice expressing humanised VCP with the dominant mutation A232E (mVCP), which manifest the characteristic pathological features of IBM [32, 33]. Treatment of mVCP mice with arimoclomol not only reduced protein aggregation in muscle fibres of mVCP mice, but also reduced TDP-43 mislocalisation, myofibre atrophy and degeneration. Importantly, these improvements in IBM-like pathology in muscles of mVCP mice were reflected by significant improvements in muscle function, including increased force generation. These beneficial effects of arimoclomol in mVCP mice are likely to result, at least in part, from an increase in the expression of HSPs, as a twofold increase in the expression of HSP70 was observed in muscles of treated mVCP mice compared to that of untreated mVCP mice [33]. These findings were instrumental in advancing arimoclomol to a randomised, double-blinded and placebo-controlled, proof-of-concept trial for the treatment of sporadic IBM, which concluded that arimoclomol was safe and well tolerated in patients, with exploratory efficacy data showing trends towards improvement in physical function and muscle strength in the arimoclomol-treated group [33]. These findings further led to a phase 2/3 efficacy study of arimoclomol for the treatment of sporadic IBM (ClinicalTrials.gov Identifier: NCT02753530). Furthermore, a double-blind, placebo-controlled safety and tolerability trial of arimoclomol in patients with rapidly progressive *SOD1* ALS (which also recorded preliminary efficacy data) showed that arimoclomol is safe and well-tolerated, and although not powered for therapeutic effect, the results of the efficacy outcome measures suggested a possible therapeutic benefit of arimoclomol [34]. Additional clinical and preclinical studies have demonstrated that arimoclomol crosses the blood brain barrier and is well tolerated [35–37]. Based on these findings, a Phase 3, randomised, placebo-controlled trial of arimoclomol in ALS was undertaken and recently completed (ClinicalTrials.Gov Identifier: NCT03491462). Although outcome targets were not found to be met in the non-stratified patient population, forthcoming post hoc analysis of the trial data may indicate patient sub-groups which responded to the treatment (personal communication with industry partner Orphazyme ApS). Arimoclomol, recently acquired by KemPharm Inc, has now gained FDA designation as a breakthrough therapy for Niemann-Pick disease type C (NPC), a lysosomal storage disorder which affects several organs including the brain.

Since mVCP mice have been reported to also display spinal cord and brain pathology which is similar to that observed in MSP, and which reflects ALS and FTD [32], respectively, we used this mouse model to examine the potential beneficial effects of augmenting the HSR on ALS and FTD-like pathology. We observed a significant improvement in the pathology present in both the spinal cord and brain of mVCP mice following treatment with arimoclomol. Furthermore, we also examined the effect of upregulating the HSR by arimoclomol treatment in human cellular models, including mutant VCP patient fibroblasts and iPSC-derived motor neurons from ALS patients with VCP mutations, which showed arimoclomol rescues key degenerative features in these mVCP patient cells in vitro. Importantly, the pathological features that were improved by arimoclomol in mVCP mice and mVCP patient cells were also found to be present in in post-mortem brain tissue from patients with FTD, confirming the relevance of the findings in our experimental models. These results provide further evidence that amplification of the HSR may be a beneficial therapy for both non-SOD1 ALS, as well as FTD.

## Materials and Methods

### Breeding and Maintenance of Mutant VCP Mice

All experimental work was carried out under licence from the UK Home Office (Scientific Procedures Act 1986) and was approved by the Animal Welfare and Ethical Review Board of UCL Institute of Neurology. Transgenic mice overexpressing the wild-type or mutant (A232E) human VCP gene under the cytomegalovirus (CMV)-enhanced chicken beta-actin promotor were generated at St Jude Children’s Research Hospital, Memphis, TN, USA [32] and a colony established and maintained at UCL, UK. These mice had been repeatedly backcrossed to C57-Black-6J mice. The colony was increased by breeding transgenic VCP female mice to C57Bl/6J wildtype males. Only male offspring were used in this study to prevent sex differences confounding the data. Offspring were genotyped by PCR of tail DNA.

All mice used in this study were housed in a temperature and humidity-controlled environment maintained on a 12-h light/dark cycle. Food and water were provided ad libitum. In these experiments, wildtype human VCP (wtVCP) mice were used as a transgenic control for the mutant human VCP (mVCP) mice.

### Arimoclomol Treatment Regime

Wildtype and mutant VCP mice were treated with either arimoclomol (obtained from Orphazyme ApS.) or vehicle (water). Following genotyping, mice were randomly divided into the following treatment groups: (i) Non-transgenic wildtype mice treated with water alone (WT); (ii) Transgenic wildtype mice treated with water alone (wtVCP); (iii) A232E mutant VCP mice with water alone (mVCP); (iv) A232E mutant mice treated with arimoclomol (mVCP+A). Mice were weighed fortnightly to adjust arimoclomol dosage at 120mg/kg given in drinking water. In pilot studies, the average volume of drinking water consumed per 24 h by mice was established. The concentration of arimoclomol required per cage was calculated according to the weight of each mouse in the cage and the highest value taken to achieve the required dose of 120mg/kg/day per mouse (minimum). This concentration was then made up in 50ml bottles using the appropriate volume of arimoclomol and tap water. Mice were treated from 4 months of age (start of symptomatic stage) to time of examination at 14 months.

### Assessment of Motor Unit Number

For in vivo electrophysiology experiments, mice were deeply anaesthetised with 1.5–2.0% isoflurane in oxygen delivered through a Fortec vaporiser (Vet Tech Solutions Ltd.). The distal tendon of the extensor digitorum longus (EDL) muscles in both hindlimbs were exposed and dissected free of other tendons before being attached by silk thread to isometric force transducers (Dynamometer UFI Devices, Welwyn Garden City, UK). The sciatic nerve was exposed and sectioned, and all branches were cut except for the deep peroneal nerve that innervates the EDL muscles. Isometric contractions were elicited by stimulating the sciatic nerve using square-wave pulses of 0.02-ms duration at supramaximal intensity, using silver wire electrodes. The number of motor units in the EDL muscles was assessed by stimulating the motor nerve with stimuli of increasing intensity, resulting in stepwise increments in twitch tension because of successive recruitment of motor axons. The resulting traces were counted to obtain total number of motor units. Force transducers were connected to a PicoScope 3423 oscilloscope (Pico Technology) and subsequently analysed using PicoScope software v5.16.2 (Pico Technology). All experiments were carried out at room temperature (approx. 23°C). Animals per group; *n*=10 WT, *n*=10 wtVCP, *n*=13 mVCP, *n*=10 mVCP+ arimoclomol. For all animals, both hindlimbs were assessed. All animals were assessed at 14 months of age.

### Histochemistry

Following administration of terminal anaesthesia (pentobarbitone injection) mice underwent transcardiac perfusion with 4% paraformaldehyde. The lumbar region of the spinal cord and the complete brain were removed, and 20-μm transverse sections were cut. For motor neuron counts sections were stained with gallocyanine (a Nissl stain). Motor neurons located within the sciatic motor pool, in which the nucleolus was visible, were counted in each ventral horn on every third section between L2 and L5 levels of the spinal cord. *n* = 5 animals per experimental group.

For immunofluorescent labelling, frozen sections were blocked for 1 h at room temperature in blocking solution (10% normal goat serum in phosphate-buffered saline (PBS) + 0.1% Triton X-100), followed by incubation with primary antibodies against the C-terminal of TDP-43 (ProteinTech, 12892-1-AP Rabbit polyclonal 1:400), ubiquitin (GeneTex, GT7811 Mouse monoclonal 1:500), HSP70 (Santa Cruz, W27 Mouse monoclonal 1:100), GFAP-Cy3 (Sigma, G-A-5 Mouse monoclonal 1:1000), β-III tubulin (Cambridge Bioscience, 3525-100 Rabbit polyclonal 1:100 or Thermo Fisher 236-10501 Mouse monoclonal 1:100), Tia1 (Abcam, ab205063 Rabbit polyclonal 1:50), p62 (Abcam, ab56416 Mouse monoclonal 1:200), for 1 h at room temperature. Sections were washed in PBS and incubated for 2 h at room temperature with the appropriate fluorescently labelled secondary antibodies. 4′, 6-Diamidino-2-Phenylindole (DAPI; Sigma, 1:1000) incubation to label nuclei or Fluoromyelin Red myelin stain (Thermo Fisher, F34652 1:300) to label myelin was performed for up to 1 h. Sudan black was applied to sections for 10 min to quench autofluorescence prior to coverslip mounting. Brain and spinal cord sections from three mice per experimental groups were assessed for each antibody tested and compared to negative controls run simultaneously.

For DAB (3, 3′-diaminobenzidine) staining, sections were washed in PBS 0.1% Triton X + 3% hydrogen peroxide before being blocked in 5% normal goat serum in PBS. Sections were incubated overnight with primary antibodies p62 (Abcam, ab56416 mouse monoclonal 1:400) or LC3 (Novus biologicals, NB100 Rabbit polyclonal 1:1000) in PBS with 3% normal goat serum. Following a wash in PBS 0.1% Triton X, sections were incubated for 2 h in anti-mouse or anti-rabbit HRP secondary antibodies (Jackson ImmunoResearch, 1:100). An avidin-biotin conjugate layer was made up using VECTASTAIN ABC system (Vector labs) following manufacturer’s instructions and DAB solution produced using a DAB substrate kit (Vector labs) following manufacturer’s instructions. DAB solution was applied to sections until a colour change was observed before rinsing sections in PBS. DAPI was used to label nuclei. Sections were dehydrated in ethanol and cleared using Histo-clear.

Fluorescent and bright-field images were visualised under a Leica fluorescent microscope and analysed using Leica Application Suite software (Leica Microsystems, Germany).

### Assessment of Motor Neuron Soma Area 

Spinal cord sections (20-μM thickness) from 3 animals per experimental group were stained with gallocyanine. Ten images of spinal cord regions L4 and L5 were taken at ×20 magnification. Motor neurons from sciatic pool of left and right ventral horn in each section were drawn around to assess the total area of each cell using the Leica Application Suite software (Leica Microsystems, Germany).

### HSP70 Intensity Measurement 

Spinal cord ventral horn sections of 20-μm thickness were fluorescently labelled for β-III tubulin and HSP70. Images from 3 animals per experimental group were taken using a Leica DMR fluorescent microscope under the same microscope and image exposure settings, at ×40 magnification. Motor neurons were identified by expression of β-III tubulin as described above (labelled with Alexa Fluor 568, red secondary antibody). Using Image J software, each β-III tubulin-labelled neuron which was clearly and completely visible was drawn around to generate a ‘region of interest’. This region of interest was subsequently duplicated in the corresponding HSP-70-labelled image from which the total cell fluorescent intensity (for HSP70 labelled with Alexa Fluor 488, green secondary antibody) per cell was measured, correcting for background fluorescence and cell area. Number of cells per experimental group assessed are as follows; wtVCP = 215, mVCP = 219 and mVCP + arimoclomol = 215. Approximately 10 randomly selected fields of view per animal were taken, and the corrected total cell fluorescent (CTCF) intensities were generated from the mean per group, relative to wtVCP controls.

### Western Blots

Tissue samples were homogenised in RIPA (radioimmunoprecipitation assay) buffer (2% SDS, 2 mM EDTA, 2 mM EGTA in 5mM Tris, pH 6.8) and spun at 14,000 rpm for 15 min to pellet the debris.

Protein concentration was determined using a colorimetric protein assay system according to manufacturer’s instructions (Bio-Rad Laboratories). Plates were incubated for 15 min at room temperature before absorbance was measured at 750 nm on a spectrophotometer.

Homogenised samples in Laemmli sample buffer were loaded on acrylamide gels and run for 1 h. Proteins were transferred onto a nitrocellulose membrane (Amersham).

Blots were blocked in TBS+ 0.1% Tween 20+ 5% bovine serum albumin for 1 h at room temperature before incubating overnight at 4°C with primary antibody anti-HSP70 (Santa Cruz W27, Mouse monoclonal) at 1:1000 dilution. Actin (Abcam ab8226 Mouse monoclonal, 1:2000 dilution) or α-tubulin (Sigma-Aldrich DM1A Mouse monoclonal, 1:2000 dilution) were used as loading controls. Membranes were washed in either PBS+ 0.1% TWEEN or TBS+ 0.1% TWEEN and then incubated in HRP-conjugated secondary antibodies (Dako, 1:1000; Thermo Fisher, 1:500) for 2 h at room temperature. StrepTactin (Bio-Rad; 1:10,000) was also added for visualisation of the protein ladder. Blots were visualised using Supersignal chemiluminescent HRP substrate (Thermo Fisher). Densitometry was analysed using ImageJ software (National Institutes of Health, Bethesda, MD, USA). Band intensities for the samples were normalised against that of the loading control in each blot.

### VCP Patient Fibroblast Culture

Disease causing mutations in VCP are very rare, resulting in a limited availability of patient samples. However, for this study, we were able to obtain four mVCP patient fibroblast lines (mutations R155H or R93C) and three age-matched healthy control lines from Professor Hanns Lochmüller at the MRC Centre Neuromuscular Biobank (Newcastle University). Collection of samples from patients and healthy individuals and their use in research have been ethically approved by the ‘Newcastle and North Tyneside 1 Research Ethics Committee’ with REC reference number 08/HO906/28 + 5 with signed written consent obtained from patients.

Fibroblasts were grown in tissue culture flasks or 24-well plates in fibroblast media (10% fetal bovine serum and 2% PenStrep in Dulbecco’s Modified Eagle Medium (DMEM) GlutaMAX-I), which was changed every 2–3 days. Cells were sub-cultured upon reaching 90% confluence using standard sub-culture and storage techniques. All cells for experiments were used at passage 2–6 and maintained at 37°C with 5% CO_2._

### Arimoclomol Treatment of Fibroblast Cultures

When cells reached ~60% confluency in 24-well plates, cultures were treated with 10–400μM arimoclomol dissolved in PBS (Invitrogen), directly added into media for 24 h. Control cells were left untreated.

### Fibroblast Immunocytochemistry

Fibroblasts were cultured on glass coverslips in 24-well plates and upon reaching approximately 70% confluency, fixed with 4% paraformaldehyde (PFA). Following fixation, cells were incubated for 1 h at room temperature with 10% normal goat/donkey serum in 0.1% PBS-Triton-X100 followed by overnight incubation at 4°C with primary antibody against TDP-43 (ProteinTech 12892-1-AP Rabbit polyclonal 1:500). Phalloidin Alexa Fluor™ 488 (Thermo Fisher, 1:200) was used to visualise actin cytoskeleton of fibroblasts. One-hour incubation with fluorescently labelled secondary antibody goat/donkey anti-rabbit/ mouse Alexa Fluor 568 (Invitrogen, 1:500) followed. Nuclear marker DAPI (1:2000) was added for 15 min before coverslips were mounted onto glass slides. Negative controls omitting primary antibodies were carried out in parallel for all experiments.

Fluorescent images were visualised under a Leica DMR microscope and analysed using Leica Application Suite software (Leica Microsystems, Germany).

### Disrupted Nuclei Counts

To assess the percentage of cells with morphological nuclear abnormalities, fibroblasts were stained with DAPI to outline the nuclear region of fibroblasts. A series of ten images were taken at x20 magnification, from one coverslip for each control or patient cell line, using a Leica DMR microscope. The number of cells with nuclear abnormalities were manually counted using ImageJ and the percentage of the total number of cell nuclei calculated. Counts in approximately 400–800 cells were undertaken in each of the control and patient cell lines. This was repeated in three separate experiments. All experiments were undertaken blind to experimental group.

### iPSC Culture and Motor Neuron Differentiation

iPSC cultures represent four clonal lines from 3 mVCP patients with R155C or R191Q mutations and four healthy controls. iPSCs were maintained on Geltrex (Life Technologies) with Essential 8 Medium medi (Life Technologies), and passaged using EDTA (Life Technologies, 0.5mM). All cell cultures were maintained at 37°C and 5% carbon dioxide. Motor neuron (MN) differentiation was carried out using a previously published protocol (Hall et al., 2017). Briefly, iPSCs were first differentiated to neuroepithelium by plating to 100% confluency in chemically defined medium consisting of DMEM/F12 Glutamax, Neurobasal, L-glutamine, N2 supplement, non-essential amino acids, B27 supplement, β-mercaptoethanol (all from Life Technologies) and insulin (Sigma). Treatment with small molecules from day 0–7 was as follows: 1μM Dorsomorphin (Sigma), 2μM SB431542 (Sigma), and 3 μM CHIR99021 (Sigma). At day 8, the neuroepithelial layer was enzymatically dissociated using dispase (GIBCO, 1 mg/ml), plated onto Geltrex coated plates and next patterned for 7 days with 0.5 μM retinoic acid and 1 μM Purmorphamine. At day 14, spinal cord motor neuron precursors were treated with 0.1 μM Purmorphamine for a further 4 days before being terminally differentiated in 0.1 μM Compound E (Sigma) to promote cell cycle exit. Cells were treated with 50 μM arimoclomol 5 days after terminal differentiation and fixed with 4% PFA after a further 24 h.

The immunostaining protocols used for iPSC derived motor neurons were the same as that for fibroblasts. Additional primary antibodies used for iPSC cultures were ubiquitin (GeneTex GT7811 Mouse monoclonal 1:500), p62 (Abcam ab56416 Mouse monoclonal 1:100), HSP70 (Santa Cruz Mouse monoclonal 1:100) and β-III tubulin (TUJ1, Cambridge Bioscience 3526-100 Rabbit polyclonal 1:100). Additional secondary antibody used for iPSC cultures was goat/donkey anti-rabbit/mouse Alexa Fluor 488 (1:500; Invitrogen). Number of cells with ubiquitin positive aggregates were quantified from 5 images per genotype/treatment from 3 separate experiments.

### Post-mortem FTD Tissue

Post-mortem brain tissue was obtained from the Queen Square Brain Bank for Neurological disorders, UCL Institute of Neurology, Wakefield Street, London WC1N 1PJ. Frozen cortical sections and frozen tissue blocks were obtained from four patients with FTD and three healthy controls. See Supplementary Table 1 for further information.

### Statistical Analysis

All data are presented as mean ± SEM. Analyses were performed using GraphPad Prism analysis software to determine the presence of statistically significant differences (*p*<0.05) using unpaired *t* tests, one-way or two-way analysis of variance (ANOVA) as appropriate. For motor neuron counts, experimental groups were compared using ANOVA test with Tukey’s post hoc analysis. All error bars on bar charts and graphs represent SEM. The investigator was blind to experimental conditions of each animal when undertaking the physiological recordings of motor unit number, and morphological analysis of mouse tissue, including assessment of motor neuron survival and soma area measurements from spinal cord tissue. For disrupted nuclei counts, experimental groups were compared using a two-way ANOVA with Tukey’s all pairwise multiple comparisons post hoc analysis. For TDP-43 nuclei intensity measurements, experimental groups were compared using a two-way ANOVA with Tukey’s post hoc analysis.

## Results

### Amplification of the HSR Improves the ALS Phenotype of mVCP Mice

**Fig. 1:**
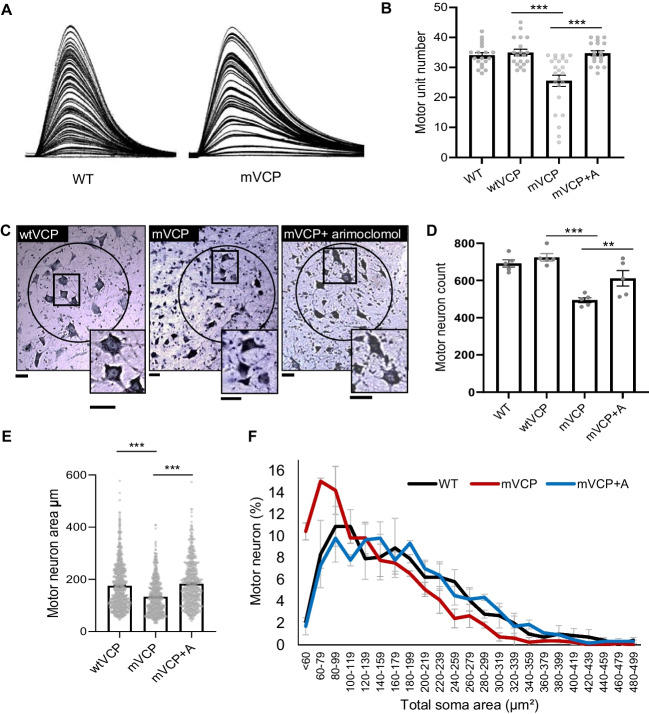
Loss of motor neurons and motor units in mVCP mice is improved with arimoclomol treatment. (**A**) Examples of isometric twitch force traces of the EDL muscle from representative non-transgenic wildtype (WT) control and mVCP mice. Each increment represents recruitment of a motor unit with increasing nerve stimulation. (**B**) Bar chart shows quantification of motor unit number in all experimental groups at 14 months of age. *** *p*=0.0002 (one-way ANOVA, *n*=10 WT, *n*=10 wtVCP, *n*=13 mVCP, *n*=10 mVCP+ arimoclomol mice per group, both hindlimbs assessed). (**C**) Nissl-stained images of spinal cord sections from the L4 region of wtVCP, mVCP and arimoclomol-treated mVCP mice at 14 months of age. Sciatic pool neurons are circled. Insets show higher magnification images. Scale bars = 20μm. (**D**) Bar chart showing number of motor neurons present in the spinal cord sciatic pool from a means of all animals per experimental groups. *p=0.029, ***p=0.0001 (one-way ANOVA, *n=*5 animals per group). (**E**) Bar chart representing the mean motor neuron area across cohorts. ***p=0.0001 (one-way ANOVA, *n*=3 animals per group). (**F**) Size distribution graph by total somal area (μm^2^) of sciatic pool motor neurons from WT, mVCP and arimoclomol-treated mVCP mice (from 10 images of spinal cord regions L4 and L5, one-way ANOVA, *n*=3 mice per group)

The loss of functional motor units is the defining characteristic of ALS and accounts for the loss of innervation at the neuromuscular junction and the resulting muscle paralysis. We have previously reported that mice expressing the human transgene for mutant VCP (A232E) have significant muscle pathology and reduced muscle strength [33]. As shown in Fig. 1, electrophysiological analysis of 10–13 mice per experimental group of the same mVCP A232E strain revealed that these mice at 14 months of age also manifest significant motor deficits, reminiscent of ALS [27]. Thus, compared to control mice expressing wild-type human VCP (wtVCP), there was a 26.5% (*p*=0.0002) reduction in the number of functional motor units innervating the hindlimb extensor digitorum longus (EDL) muscle in mVCP mice (Fig. 1A, B), decreasing from an average of 34 ± 1.5 motor units in wtVCP mice to 25 ± 2 in mVCP mice.

Amplification of the HSR in mVCP mice by daily treatment with 120mg/kg of arimoclomol, from 4 months (symptom onset) to 14 months of age, resulted in a complete prevention of motor unit loss in EDL muscles. In the arimoclomol-treated mVCP cohort, 35 ± 1.9 motor units innervated the EDL muscle (*p*=0.0002), which is similar to the number in both wtVCP mice (34 ± 1.5) and WT non-transgenic littermate controls (34.3 ± 0.9; Fig. 1B). There was no significant difference in the number of EDL motor units between the two control groups wtVCP and non-transgenic WT littermates.

Following these acute physiological experiments, the spinal cord and brain were removed for histopathological analysis. Quantification of the mean number of motor neurons in the sciatic motor pool, which innervate hind limb muscles, showed no significant difference in the number of motor neurons in wtVCP and non-transgenic WT controls, in which there were 725 ± 20 and 692 ± 20 motor neurons, respectively. However, there was a significant decrease in motor neuron survival in mVCP mice, in which only 494 ± 14 motor neurons survived; this represents a 32% reduction in motor neuron survival compared to wtVCP controls (*p*=0.0001, Fig. 1C, D). Amplification of the HSR by treatment with arimoclomol significantly improved motor neuron survival in mVCP mice, and 612 ± 42 motor neurons survived, an improvement of 24% (*p*=0.029) when compared to untreated mVCP mice.

Furthermore, in untreated mVCP mice, motor neurons, which survived at 14 months, had an abnormal morphology, with small, compacted cell bodies, contrasting with the large polygonal shape of motor neurons observed in control animals (Fig. 1C). We therefore assessed the total soma area of motor neurons in the sciatic pool in each cohort of mice (*n*= 3 per group). As can be seen in Fig. 1E, there was a clear reduction in the mean soma size of the motor neurons that survived in mVCP mice compared to wtVCP controls. Size distribution analysis revealed that the reduction in mean motor neuron soma area in mVCP mice was predominantly due to the preferential loss of large, likely alpha motor neurons, and an increase in the proportion of smaller motor neurons (Fig. 1F). This shift in the size distribution of motor neuron soma size in mVCP mice was prevented in arimoclomol-treated mVCP mice, in which the motor neuron soma size distribution was similar to that observed in normal WT mice (Fig. 1F).

**Fig. 2:**
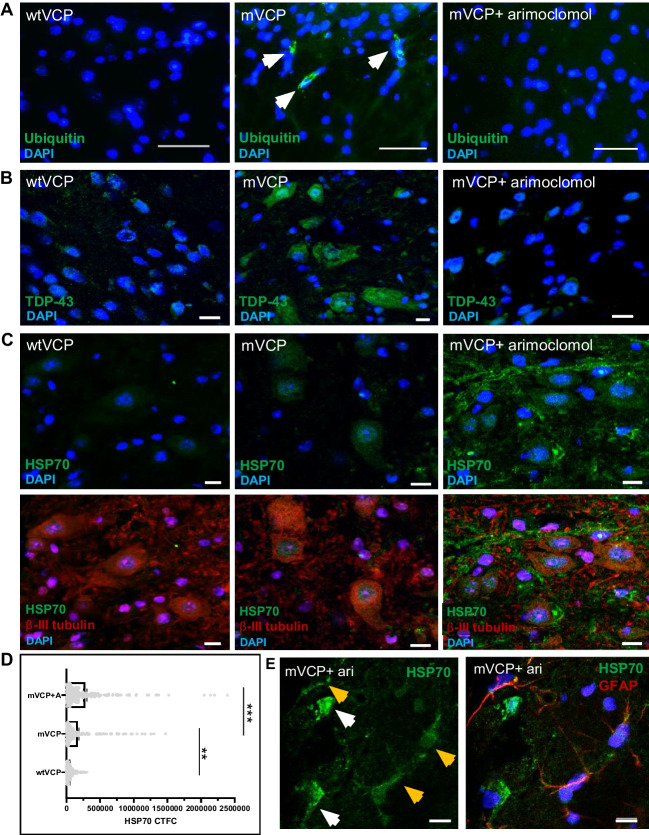
Ubiquitin and TDP-43 pathology in mVCP mouse spinal cord is improved with arimoclomol and is associated with increased HSP70. Immunofluorescent images of lumber spinal cord sections from wtVCP, mVCP and arimoclomol-treated mVCP mice showing, (**A**) ubiquitin immunoreactivity in neurons (arrows indicate ubiquitin-positive protein aggregates), (**B**) TDP-43 localisation in neurons and, (**C**) HSP70 expression in spinal cord sections with and without neuronal marker (β-III tubulin, red). Nuclei labelled with DAPI (blue). (**D**) Bar graph representing the corrected total cell fluorescence intensities (CTCF) of HSP70 in immunolabelled spinal cord ventral horn motor neurons from each experimental group (average 216 neurons per group, one-way ANOVA, **p=0.0008, ***p= 0.0001). (**E**) Example image of HSP70 expression in GFAP co-labelled spinal cord sections with and without the glial marker from an arimoclomol-treated animal. White arrows show GFAP-negative neuronal cells; yellow arrows show GFAP-positive glial cells. DAPI labels nuclei (blue). Scale bar = 10μm

Aggregation of misfolded proteins in ubiquitin-containing inclusions and cytoplasmic mislocalisation of the nuclear RNA-binding protein TDP-43 are key hallmarks of ALS pathology. We observed no ubiquitin pathology in the spinal cord of wtVCP mice or non-transgenic WT littermates at 14 months of age. In contrast, ubiquitin-positive protein aggregates were detected in motor neurons of mVCP mice (Fig. 2A). Whilst immunostaining for cytoplasmic TDP-43 was low in wtVCP tissue, a distinct increase in cytoplasmic TDP-43 was observed in motor neurons of mVCP mice (Fig. 2B). In contrast, in mVCP mice treated with arimoclomol, cytoplasmic TDP-43 mislocalisation and ubiquitin pathology were similar to that seen in control animals (Fig. 2B).

HSP70 levels in spinal motor neurons cord were quantified to better understand the HSR response in these cells directly. The pathological changes observed in the spinal cord of mVCP mice were associated with an almost 3-fold increase in the expression of HSP70 in motor neurons, compared to that in wtVCP control animals in which there was no significant pathology (Fig. 2C, D). Importantly, this indicates that the endogenous response to cell stress has been triggered in the mVCP motor neurons, although this was not sufficient to prevent the development of pathology. Amplification of the HSR in mVCP mice by treatment with arimoclomol resulted in approximately a 4-fold increase in HSP70 compared to controls (Fig. 2C, D). In addition, in arimoclomol-treated mVCP mice, HSP70 levels were also enhanced in non-neuronal cells, initially seen as filamentous-like structures negative for β-III tubulin (Fig. 2C), which subsequent GFAP-labelling confirmed to be astroglia (Fig. 2E). This likely reflects an additional cytoprotective response to mVCP-induced stress in the spinal cord.

**Fig. 3:**
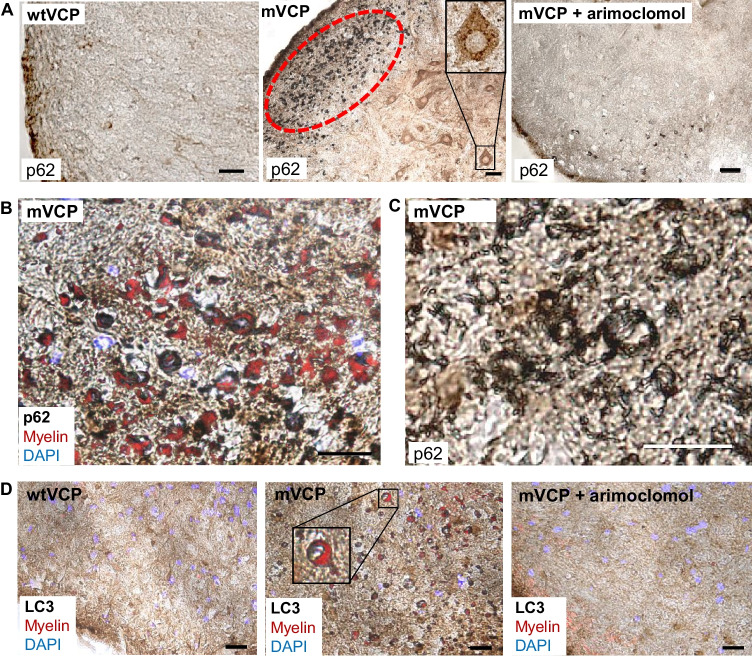
Increased expression of p62 and LC3 in the spinal cord grey and white matter of mVCP mice is reduced with arimoclomol treatment. (**A**) Representative histological images of p62 expression in the spinal cord of wtVCP, mVCP and arimoclomol-treated mVCP mice. mVCP spinal cord shows increased p62 expression in white and grey matter and aggregated p62 in sciatic pool motor neurons (inset, high magnification image) and oligodendrocytes (ringed in red). (**B**) High magnification image of increased p62 expression with myelin shown in red. (**C**) High magnification image of collapsed myelin sheath in mVCP spinal cord white matter. (**D**) Immunohistochemistry showing LC3 expression in mouse spinal cord from wtVCP, mVCP and mVCP mice treated with arimoclomol, co-localised with myelin (red). Inset shows higher magnification image of an axon surrounded by myelin (red). Scale bar = 10μm

VCP is known to play an essential role in autophagy [13], and dysfunctional autophagy has been implicated in the pathogenesis of ALS and MSP. For example, MSP patients expressing mVCP show evidence of disrupted autophagy, with accumulation of the key autophagic markers p62 (Sequestosome 1) and LC3 within myofibres [13, 38]. We therefore examined the pattern of expression of these two autophagic markers in the spinal cord of mVCP mice at 14 months of age. We observed an increase in p62 expression in both the white and grey matter of the spinal cord compared to control animals (Fig. 3A–C), with p62 aggregates clearly visible in motor neurons (Fig. 3A—magnified inset in mVCP image). Co-labelling with fluoromyelin suggested that the intense p62 staining observed in mVCP spinal cord sections was associated with oligodendrocytes (Fig. 3B). Closer examination of the p62-positive oligodendrocytes revealed gross myelin disruption around axons, deviating from the classic ‘onion bulb’ structure of healthy myelin, suggestive of axonal and/or myelin degeneration (Fig. 3C). This pattern of p62 expression was not observed in spinal cords from any control animals and was visibly reduced in arimoclomol-treated animals.

We also examined the expression pattern of the autophagosome marker LC3. LC3 is recruited to the autophagosomal membrane during autophagy and later degraded in the autolysosomal lumen and, as such, is routinely used as a marker of autophagic activity in cells [39, 40]. In mVCP mice we observed an increase in the expression of LC3 in the spinal cord and in particular in oligodendrocytes associated with abnormal myelination (Fig. 3D). Together with the accumulation of p62, this provides further evidence of defective autophagy in these cells due to the presence of mutant VCP. Importantly, the accumulation of p62 and LC3 and abnormal myelination was visibly reduced in mVCP mice in which the HSR was amplified by arimoclomol treatment.

### Amplification of the HSR Improves the FTD Phenotype in the Brain of mVCP Mice

A third of patients diagnosed with MSP caused by mutations in VCP develop FTD [9], and mutations in VCP cause <1% of all FTD cases [41]. To determine whether mVCP mice exhibit FTD-like pathology, we next examined their brain.

**Fig. 4:**
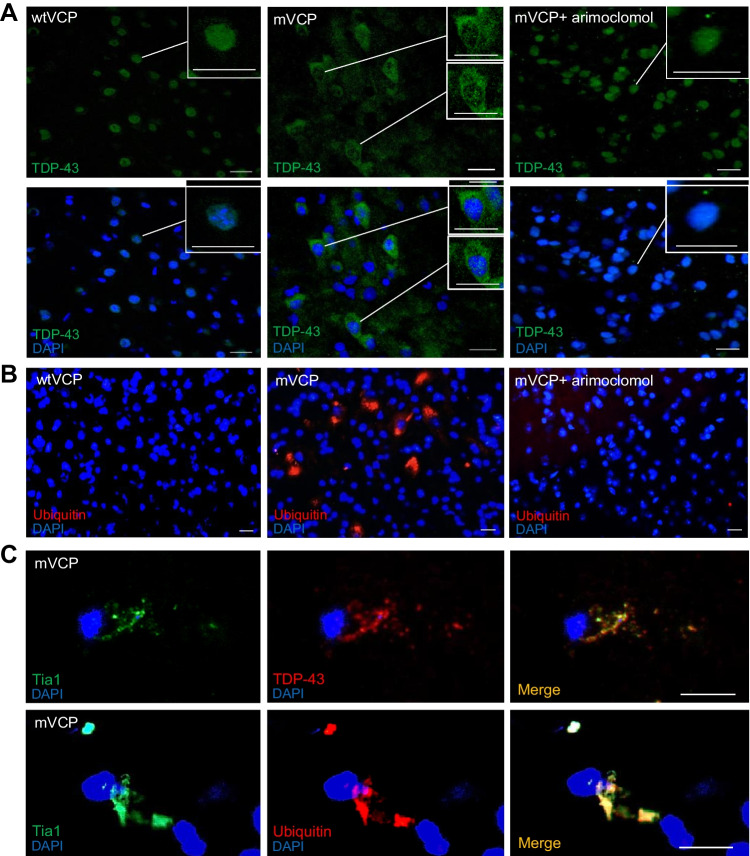
TDP and ubiquitin pathology in mVCP mouse brain is improved with arimoclomol treatment. (**A**) TDP-43 localisation in cortical cells. Insets show cells at higher magnification. Scale bar = 20μm. (**B**) Ubiquitin immunoreactivity in mouse cortex shows ubiquitin-positive aggregates in mVCP brain (red). Scale bar = 20μm. (**C**) Stress granule marker Tia1 colocalised with TDP-43 and ubiquitin in mVCP brain. Scale bar = 10μm. DAPI labels nuclei (blue) in all images

Similar to ALS and MSP, cytoplasmic mislocalisation of TDP-43 is a pathological characteristic of FTD, with mislocalised TDP-43 present in the brain of approximately 50% of FTD cases, and brain pathology often indistinguishable from that seen in FTD patients with a mutation in the *TARDBP* gene itself [9, 42]. Cytoplasmic TDP-43 is often found either dispersed in the cytosol or within inclusion bodies, concomitant with its nuclear clearance [43], indicating that a loss of normal nuclear function as well as gain of toxic cytoplasmic function may play a role in disease pathogenesis [42]. In the brain of 14-month-old mVCP mice, we observed an increase in the number of cortical neurons with distinct cytoplasmic TDP-43 mislocalisation compared to control mice, with many neurons showing nuclear clearance of TDP-43 (Fig. 4A). In contrast, TDP-43 expression in the cortex of arimoclomol-treated mVCP mice was similar to control animals, and no cytoplasmic mislocalisation associated with nuclear clearance of TDP-43 was apparent in the brains of the arimoclomol-treated group (Fig. 4A).

We next examined the expression of ubiquitin in the brain of 14-month old mVCP mice. Similar to our findings in the spinal cord, in mVCP mice we observed ubiquitin-positive intracellular aggregates in the brain, although these were not limited to the cortex. No ubiquitin-positive aggregates were detected in control animals, or in mVCP mice treated with arimoclomol (Fig. 4B).

Microtubule-associated protein tau (MAPT) is associated with a well-known genetic form of FTD, and deposits of p-tau are often detected in post-mortem brain of dementia patients [44]. We found extracellular deposits of phosphorylated tau (p-tau, AT8) in the brain of mVCP mice (Supplementary Fig. 1A) which may have formed through non-specific aggregation of proteins. The tau deposits were often observed surrounded by Iba1-positive microglia or GFAP-positive astrocytes, suggesting an inflammatory response to the abnormal presence of p-tau (Supplementary Fig. 1A). In contrast, in control and mVCP mice in which the HSR was amplified by arimoclomol, no tau-positive deposits were observed in any area of the brain assessed.

Whilst TDP-43 plays an important role in RNA metabolism, mislocalisation of this protein suggests that other RNA-associated proteins may also be affected in mVCP mice. Stress granule formation is a highly evolutionarily conserved cytoprotective mechanism to temporarily store stalled translation pre-initiation complexes during episodes of cellular stress [45]. We examined the expression of Tia1, an RNA-binding protein, known to be present in stress granules [46] in mouse brain tissue. We discovered that Tia1 co-localised with both TDP-43 and ubiquitin inclusions in the cytoplasm of mVCP brains suggesting the possible formation of non-specific protein aggregation (Fig. 4C). It has been demonstrated in vitro that TDP-43-containing granules in the cytoplasm may seed aggregation through RNA binding [47, 48]. Furthermore, regenerating myofibres reportedly contain TDP-43 ‘myo-granules’ which may be the precursors for aggregation in diseased muscle, as shown in mutant VCP (A232E) mouse muscle [47]. It is therefore possible that a similar phenomenon also occurs in the brain of mVCP mice, leading to the aggregates observed in this study. Two additional markers of stress granules, FMRP and G3BP, were abnormally observed only in the brain of mVCP mice (Supplementary Fig. 1B). In control animals and mVCP mice treated with arimoclomol, no aggregates positive for any of the tested stress granule markers were observed (Supplementary Fig. 1B).

**Fig. 5:**
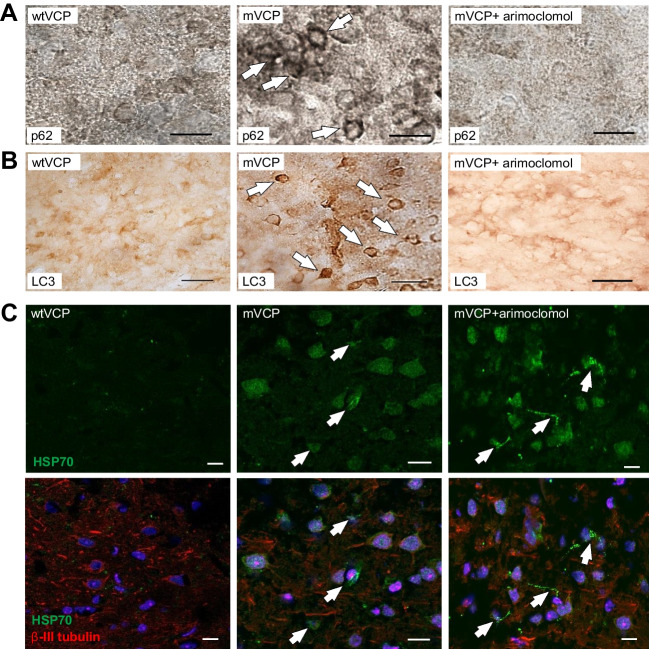
Arimoclomol treatment prevents p62 and LC3 aggregation and enhances HSP70 in mVCP mouse brain. Histological images of (**A**) p62 expression and, (**B**) LC3 expression in mouse brain sections. White arrows indicate protein aggregates. (**C**) HSP70 expression in mouse brain with and without neuronal marker (β-III tubulin, red) and nuclear marker (DAPI, blue). White arrows indicate glial cells expressing HSP70. Scale bar =10μm

Furthermore, as observed in the spinal cord, immunostaining of mVCP mouse cortex also revealed the presence of p62 and LC3-positive aggregates, with intense cytosolic LC3 staining (Fig. 5A, B). No LC3 staining was observed in control tissue or tissue from arimoclomol-treated mVCP mice.

Similar to the findings in the spinal cord of mVCP mice, brain tissue from these animals also showed an increase in the expression of HSP70 compared to wtVCP and non-transgenic WT controls, indicative of stress-induced activation of the HSR. In mVCP mice treated with arimoclomol, HSP70 expression was found to be further increased, suggesting that the HSR has been augmented by arimoclomol treatment (Fig. 5C). Interestingly, as noted in the spinal cord, HSP70 expression was significantly pronounced in glial cells in the brain, which are known to have a robust stress induced HSR.

### Pathology Observed in mVCP Mice Reflects That Observed in FTD Patient Brain Tissue

To verify whether the pathological features observed in the CNS of mVCP mice, which were improved by amplification of the HSR, have relevance to the human disease, we examined post-mortem cortical brain tissue from 4 patients diagnosed with FTD (see Supplementary Table 1 for further patient information and genetic/clinical sub-classification). A panel of the same markers used in the mVCP mice were assessed and the results compared to samples of the same region from age-matched healthy human brains.

**Fig. 6:**
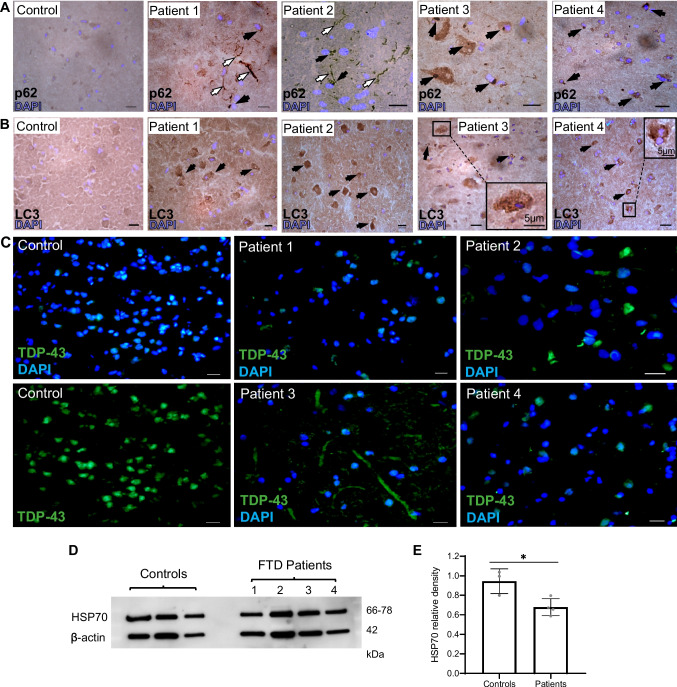
FTD patient brain pathology and HSP70 expression levels. (**A**) p62 immunohistochemistry on post-mortem brain cortex from patients diagnosed with FTD shows p62-positive inclusion bodies. White arrows indicate intensely stained neurites, and black arrows indicate cytoplasmic protein aggregates. DAPI labels nuclei (blue). Scale bar = 10μm unless otherwise indicated. (**B**) Increased LC3 expression in neurons of post-mortem brain from patients diagnosed with FTD**.** Darker stained neurons with deposits positive for LC3 observed in all FTD patients. Insets show magnification of marked regions. (**C**) Cytoplasmic TDP-43 mislocalisation was observed in all FTD patient samples (green), while rarely seen in control tissue. DAPI labels nuclei (blue). Scale bar = 10μm unless otherwise indicated. (**D**) Western blot of HSP70 expression from FTD patient brain tissue compared to healthy controls and (**E**) corresponding western blot density bar chart comparing all FTD patient HSP70 levels normalised to controls (*t* test * *p*=0.02)

Since autophagy is a common pathway that may be disrupted in FTD brain, we examined human FTD brain for the presence of autophagy-related proteins which were abnormally present in mVCP mice. Globular, juxtanuclear p62-positive cytoplasmic inclusion bodies were present in cortical brain sections of all FTD cases examined (Fig. 6A) and were similar to those seen in mVCP mice (Fig. 5A), suggesting a generalised disruption in protein homeostasis in FTD patients; no p62 immunostaining was observed in brain sections from healthy controls. Strong p62 staining was also observed in the brain of Patient 1 (FTD caused by tau mutation, Fig. 6A), similar to that reported in the brain of Alzheimer’s disease patients early in pathogenesis within neurofibrillary tangles [49]. In Patient 2 (FTD associated with mutant TDP-43), intense p62 staining was observed in neural processes in addition to cytoplasmic inclusion bodies. Interestingly, a similar pattern of TDP-43 staining has been reported in the upper cortical layers in FTD patients with a TDP-43 mutation [50]. Immunostaining of cortical sections also revealed the presence of LC3-positive aggregates in the cortical tissue of all FTD patients (Fig. 6B), similar to findings in mVCP mice (Fig. 5B) which were not seem in control sections.

Although TDP-43 mislocalisation in FTD brain is a well-established phenotype, we demonstrate extensive cytoplasmic mislocalisation of TDP-43 in all four FTD patient brains assessed, in which p62 and LC3 are also aggregated. Such mislocalisation was not observed in samples from healthy controls (Fig. 6C). Our findings thus reveal that the pathological phenotypes identified in the mVCP mouse model are indeed present in the human disease. Moreover, these phenotypes are not limited to mutations in VCP, therefore expanding the relevance of our findings of the beneficial effects of targeting the HSR to non-VCP FTD patients.

Whilst the ability to mount a HSR under conditions of cell stress is present throughout life, this cytoprotective mechanism is thought to become less effective in later life, likely contributing to the age-related increase in susceptibility to degenerative diseases [51]. In our study, compared to age-matched control post-mortem samples, HSP70 expression was indeed significantly lower in the cortex of FTD patients (Fig. 6D–E). Therapeutically augmenting this endogenous cytoprotective process may therefore be a beneficial strategy in response to neurodegenerative diseases such as ALS/FTD.

### Patient-derived Mutant VCP Fibroblasts and iPSC Motor Neurons Exhibit Pathological Characteristics Which Are Ameliorated by Amplification of the HSR

Our results show for the first time that upregulation of the HSR ameliorates the pathological deficits observed in the brain and spinal cord of mVCP mice. To further test whether the beneficial effects of this approach may have therapeutic relevance for human ALS/FTD, we next examined the effects of treatment with arimoclomol in human models of ALS/FTD by establishing patient-derived cellular models of mutant VCP.

**Fig. 7:**
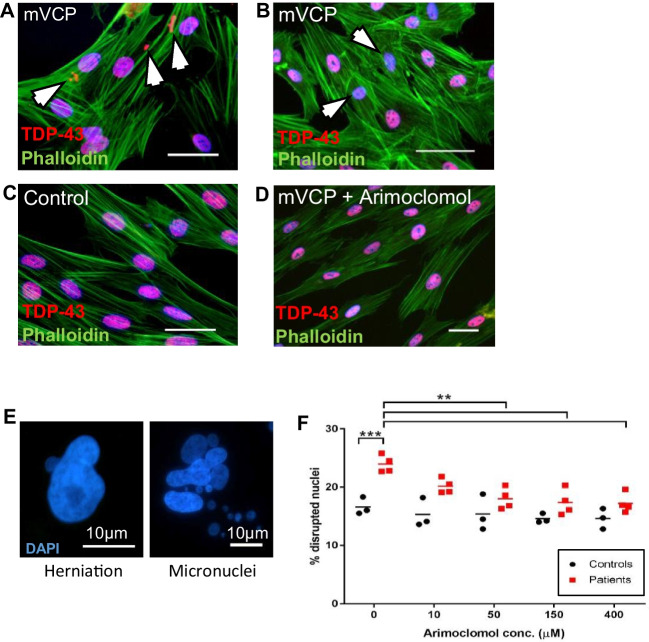
Mutant VCP patient fibroblasts exhibit pathology ameliorated by arimoclomol. Expression of TDP-43 in untreated mVCP fibroblast cultures show (**A)** TDP-43- positive aggregates or (**B**) depletion of nuclear TDP-43 (indicated by white arrows). (**C**) Control, and (**D**) arimoclomol-treated mVCP cultures show no abnormal TDP-43 expression. Scale bar = 20 μm. (**E**) DAPI-labelled fluorescent images of abnormal nuclear morphology observed in mVCP patient fibroblasts showing nuclear herniation and nuclear fragmentation generating micronuclei. (**F**) Quantification of disrupted nuclei in fibroblasts treated with increasing concentrations of arimoclomol, **p<0.01, *** *p*<0.001 (two-way ANOVA, *n*=3 controls, *n*=4 patients)

In order to investigate whether patient-derived cells can be used as a platform to test the effects of arimoclomol on VCP-relevant pathology, we initially undertook a preliminary study in mutant VCP patient fibroblasts which have been reported to display a pathological phenotype when grown in culture [52]. In mVCP patient fibroblasts, cultured from four individual patient lines (Fig. 7), cytoplasmic aggregates of TDP-43 were observed (Fig. 7A), and whilst most nuclei stained robustly for TDP-43, some nuclei stained faintly for TDP-43, indicative of nuclear depletion (Fig. 7B). This pattern of staining was observed in all patient lines. No TDP-43-positive cytoplasmic aggregation or nuclear depletion of TDP-43 was observed in control fibroblasts, in which TDP-43 immunostaining was restricted to nuclei (Fig. 7C). In arimoclomol-treated mVCP fibroblasts (10μM, 24-h treatment), the pattern of TDP-43 immunostaining was similar to that observed in healthy controls (Fig. 7D).

Furthermore, in untreated mVCP fibroblasts, we also observed a significant increase in the number of nuclei with an abnormal morphology, consisting of herniations and fragmentation of nuclei leading to the generation of micronuclei (Fig. 7E). Surprisingly, these cells were not undergoing apoptosis, as assessed by TUNEL staining for DNA double-strand breaks (Supplementary Fig. 2). Quantification of the number of aberrant nuclei in mVCP fibroblasts in the absence and presence of increasing concentrations of arimoclomol revealed a dose-dependent reduction in the number of disrupted nuclei, with a statistically significant difference observed at 50μM of arimoclomol (Fig. 7F).

Since mVCP fibroblasts successfully demonstrated the rescue of pathological features in human cells by upregulation of the HSR, we began our study in human induced pluripotent stem cell (iPSC)-derived motor neurons (iPSC-MNs) established from ALS patients expressing mutant VCP, which provide a more complex, neuronal and highly disease-specific cell culture model of neurodegeneration, and which have been previously shown to manifest a TDP-43 pathology [53].

**Fig. 8:**
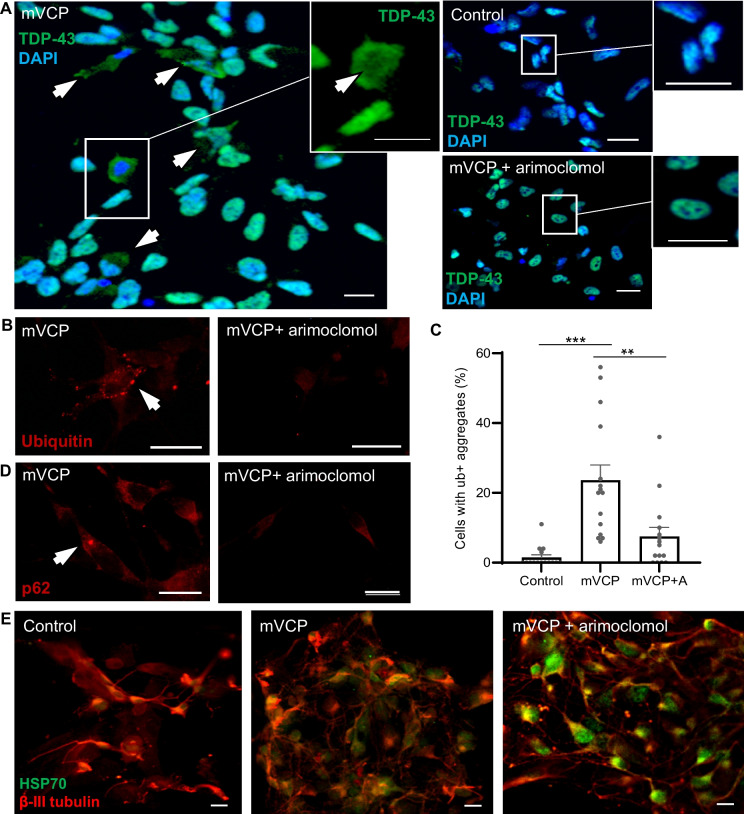
Human mVCP iPSC-derived motor neurons exhibit pathology ameliorated by arimoclomol. (**A**) TDP-43 immunoreactivity shows localisation in control, mVCP and arimoclomol-treated mVCP iPSC motor neuron cultures. Arrows indicate cells with cytoplasmic TDP-43. Magnified image of a cell in the mVCP culture with depletion of TDP-43 visible in the nuclear region (inset, arrow indicates depleted region). (**B**) Fluorescent images of ubiquitin immunoreactivity in mVCP iPSC motor neuron cultures with and without arimoclomol treatment. Arrow indicates ubiquitin-positive aggregate. (**C**) Bar chart showing percentage of cells with ubiquitin-positive aggregates **p=0.003, **p=0.0001 (one-way ANOVA, *n*=3 cultures). (**D**) p62 immunoreactivity in iPSC motor neurons from arimoclomol-treated and untreated mVCP cultures. Arrow indicates p62-positive cytoplasmic protein aggregate. (**E**) Immunofluorescent images of HSP70 expression with neuronal marker β-III tubulin. DAPI labels nuclei (blue). Scale bar = 10μm.

In mVCP patient iPSC-derived motor neurons (3 individual patient lines), we observed distinct cytoplasmic TDP-43 staining with many cells also exhibiting nuclear loss of TDP-43 (Fig. 8A, magnified image of cell in mVCP image). Importantly, mislocalised TDP-43 was rarely seen in the mVCP cultures treated with 50 μM arimoclomol and was absent from healthy control cells.

Ubiquitin-positive and p62 immuno-reactive protein aggregates were also detected in mVCP iPSC-motor neurons, either as many small bodies dispersed throughout the cell or as one large distinct globular aggregate within the cytoplasm (Fig. 8B, D). Quantification showed that there was a significant increase in the number of cells with ubiquitin-positive aggregates in mVCP neurons, from 1.5% in controls to 23.6% in mVCP cultures. In contrast, in mVCP cultures treated with arimoclomol, only 7.5% of neurons contained ubiquitin-positive aggregates (Fig. 8C). These findings corroborate our observations in mVCP mice, where abnormal ubiquitin and p62 accumulation, possibly linked to impairment of autophagy or proteasomal degradation, was present in both the spinal cord and brain. More importantly, these data clearly show that amplification of the HSR leads to a reduction in cytoplasmic ubiquitin aggregates in a specific neurological cellular system with VCP mutation.

As observed in the mVCP mice, HSP70 expression was increased in mVCP iPSC-derived motor neuron cultures under basal conditions, indicating that these cells are under stress and have activated the HSR. Treatment of mVCP iPSC-derived motor neurons with arimoclomol resulted in a clear increase in HSP70 expression above that observed in untreated mVCP iPSC-MNs (Fig. 8E), indicating an enhancement of the endogenous cytoprotective HSR, which is likely to account for the improvement in protein mishandling pathology observed in untreated cells.

## Discussion

We have previously demonstrated the beneficial effects of augmenting the HSR on the ALS phenotype of mutant SOD1 mice as well as the IBM-like muscle pathology that is present in mutant VCP mice [27, 29, 33]. In the present study, we expand these findings to examine the effects of amplifying the HSR on spinal cord and brain pathology which is also part of the MSP phenotype of mVCP mice and which we show to be similar to pathological characteristics of human ALS/FTD.

Our results show that the loss of functional motor units and corresponding degeneration of spinal motor neurons observed in mVCP mice is ameliorated by treatment with a pharmacological amplifier of the HSR called arimoclomol. In particular, large, likely alpha neurons, which were preferentially lost in mVCP mice were rescued by amplification of the HSR. The preferential degeneration of alpha motor neurons is a well-established characteristic of ALS [54, 55] and has been previously observed in the SOD1^G93A^ mouse model of ALS [27]. Importantly, this finding shows for the first time that arimoclomol is able to rescue the defining pathological hallmark of ALS not only in mouse models of SOD1-ALS [27, 29] but also in a model of non-SOD1 ALS, suggesting that it may have therapeutic potential for ALS more broadly.

We also observed distinct signs of proteotoxic cell stress in the spinal cord of mVCP mice, with the presence of ubiquitin-positive aggregates and cytoplasmic mislocalisation of TDP-43. Furthermore, we observed accumulation of p62 and LC3 in neurons and myelinating oligodendrocytes, suggesting that deficits in autophagy, in which VCP is a key player, may lead to inefficient clearance of aberrant proteins in mVCP spinal cord neurons. In addition, we observed evidence of myelin disruption in the spinal cord white matter, which may reflect axonal and/or myelin degeneration. Amplification of the HSR resulted in a significant increase in the expression of HSP70 in *both* neurons and glia and amelioration of the pathological features of ALS. These observations are consistent with previous reports demonstrating that upregulation of HSP70 improves lysosomal function and myelination in the CNS [35].

A similar pathological phenotype was observed in the brain of mVCP mice, including the presence of ubiquitinated inclusion bodies, TDP-43 mislocalisation and accumulation of p62 and LC3 in neurons; these pathological markers were again associated with an increase in the expression of HSP70. Areas of p-tau pathology were also present, with Iba1 and GFAP-positive cells surrounding p-tau lesions, suggesting the presence of an inflammatory response. Stress granule markers, co-localised with ubiquitin and TDP-43, were also found in mVCP mouse brains. This indicates that there may be impaired stress granule clearance, as previously reported when VCP function has been inhibited [56]. Amplification of the HSR by treatment with arimoclomol improved all of the pathological changes observed in mVCP mouse brains and was associated with a clear enhancement of HSP70 expression above that occurring in untreated mVCP mice in both neurons and glia. This is consistent with previous reports showing that arimoclomol readily crosses the blood brain barrier and amplifies the expression of HSP70 within the CNS [35, 37]. Upregulation of the HSR in glial cells may reflect a stress response in these cells or may be part of a neuroprotective mechanism to help defend neurons against mVCP-induced stress through the expression of heat shock proteins [57]. Glial HSP70 is known to be protective to neurons, and exogenous HSP70 has been shown to be beneficial in improving motor neuron survival in the SOD1^G93A^ mouse model of ALS [58–60]. To reduce the numbers of mice recruited to this study, only male mice were examined in this study. However, we have previously shown that the beneficial effects of arimoclomol on muscles and neurons are observed in both male and female mice in our study on the SOD1 mouse model of ALS [29], where gender differences were directly compared.

We also examined two human cellular models of mutant VCP disease, which provide more clinically relevant platforms to test the potential of HSR amplification and also allow for further analysis of some of the observations made in the mVCP mouse and FTD patient post-mortem tissues. mVCP patient fibroblast cultures demonstrated a dose-dependent improvement in abnormal nuclear morphology with arimoclomol, a pathological feature which has previously been observed in IBM patient muscle [61]. This phenomenon may be the result of altered nuclear membrane integrity brought about by an imbalance of proteins that constitute the nuclear lamina when protein homeostasis is disrupted [62].

Furthermore, arimoclomol treatment resulted in observable differences in TDP-43 pathology, thus supporting a study in human iPSC-derived motor neurons from patients with mutations in VCP.

In mVCP iPSC-derived neurons, the key degenerative phenotypes observed in mVCP mice were recapitulated, including the formation of ubiquitinated and p62-positive protein aggregates and TDP-43 pathology. Treatment with arimoclomol resulted in an improvement in these pathological features with a clear upregulation of the major chaperone protein HSP70 in these cultures.

The results in human cellular models therefore show that amplification of the HSR, by treatment with arimoclomol, ameliorates key pathological features of ALS/FTD in vitro. These findings corroborate the in vivo data from mVCP mice and demonstrate that pharmacological augmentation of the HSR is sufficient to rescue key degenerative changes in human mVCP cells in vitro.

In parallel to the experimental data, this study also confirms that the same key pathological characteristics of ALS/FTD observed in the mouse and recapitulated in the human cell models of mVCP disease, and which were ameliorated by treatment with arimoclomol, are present in cortical brain samples of FTD patients. Our results show that TDP-43 pathology was present in all FTD patient samples examined regardless of subtype diagnosed. In addition, both p62 and LC3 proteins were found to be accumulated in FTD brains as revealed by histochemistry. These findings suggest that impaired clearance of neurotoxic proteins by autophagy may be a common factor in FTD.

Although the scale, kinetics and required threshold of the HSR vary between cells and stressors, this vital mechanism routinely keeps cells free of damaged and surplus proteins [63]. As a highly adaptive system, the HSR is able to tailor the type of stress response that is most appropriate for the type of cell involved *and* for the specific type of stress they are under [64], coordinating stress-induced transcription of a variety of chaperones and co-chaperones. It has been long established that a reduction in the HSR is associated with ageing, and this may contribute to the accumulation of aberrant proteins that occurs in neurodegenerative disorders [51, 65]. All FTD post-mortem brain samples investigated in this study were from patients of relatively advanced age, ranging from 62 to 79 years old and were compared to age-matched control individuals. A reduced HSR would therefore not be unexpected these FTD patients. Pharmacologically targeting any deficiency in the ability of a cell to mount a robust HSR to stress, whether caused by aging or disease, may thus be an effective approach to alleviate neurodegenerative pathology and delay disease progression in ALS/FTD patients.

However, although arimoclomol, the specific pharmacological amplifier of the HSR examined in this study, has been shown to be effective at ameliorating disease phenotypes in multiple *preclinical* models of protein dyshomeostasis, including rodent and human cellular models, as well as in vivo, in mouse models of ALS, FTD, SBMA and IBM, these disease-modifying effects did not translate into benefits in ALS patients in a recent Phase 3 clinical trial. In view of the substantial body of biological evidence that demonstrates the effectiveness of targeting the HSR with arimoclomol, both in the current study and in previously published reports [27–30, 33, 34], it is disappointing that the recent clinical trial in ALS patients failed to meet its primary outcomes (ClinicalTrials.Gov Identifier: NCT03491462). However, clinical trial design in ALS has rarely involved patient stratification, which in a genetically heterogenous disease such as ALS may prevent identification of compounds that may have utility in sub-sets of patients at specific stages of disease progression. Indeed, when arimoclomol was tested in a double-blind, placebo-controlled safety and tolerability trial in patients with rapidly progressive *SOD1* ALS, preliminary efficacy data suggested a possible therapeutic benefit [34]. With only two drugs currently approved by the FDA and routinely used for the treatment of ALS there is an urgent need for new therapeutic agents, as Riluzole, approved in 1995, only mildly alters mortality [66], increasing lifespan by 3–6 months. A trial of the antioxidant Edaravone [67], showed improvement in the ALS Functional Rating Scale (ALSFRS), although this was not statistically significant [68]. However, in a subset of patients in whom Riluzole was used concomitantly, Edaravone showed significant efficacy as assessed by the ALSFRS and led to FDA approval [69]. The heterogeneity in treatment response has been confirmed in an exploratory meta-analysis of ALS patients treated with lithium carbonate [70]. Although the initial trial results indicated that there was no benefit in the trial participants, subsequent analysis showed a subgroup of patients homozygous for a single nucleotide polymorphism the *UNC13A* gene had a statistically significant survival benefit when treated with lithium carbonate [71]. These findings demonstrate that a heterogenous patient population may mask any therapeutic effect on subgroups of patients and that combination therapy with known disease modifiers may prove to be more effective than a single novel agent alone. Importantly, many of these limitations of trial design are now recognised, and there have been significant improvements in the design of ALS trials, including for example testing in known genetic sub-groups.

## Conclusions

The results of this study show that in both spinal cord and brain of mVCP mice and in patient cells, expression of mutant VCP gives rise to a neurodegenerative pathology that is reminiscent of ALS/FTD and MSP, including protein aggregation and TDP-43 mislocalisation. These pathological features are also observed in human FTD brain. Importantly, in mouse models and patient cells, this pathology is ameliorated by pharmacological targeting of the HSR.

Taken together with the results from our previous studies in mVCP IBM muscle, SOD1^G93A^ ALS mice [27–29] as well the AR100 mouse model of SBMA [30], our findings suggest that several common pathological characteristics develop when protein mishandling occurs in a cell, regardless of the cause, the tissue type or indeed whether murine or human. Moreover, our results demonstrate that targeting the HSR results in an amelioration of these pathological characteristics. These findings therefore support further development of compounds capable of amplifying the HSR as a therapeutic strategy for the treatment of diseases where protein dyshomeostais is a feature of disease. We await the ongoing post hoc analysis results of the recently completed Phase 3 clinical trial of arimoclomol in ALS to establish whether patient sub-populations may have been amenable to this treatment strategy. Nevertheless, the results presented here clearly show the beneficial effects of targeting the HSR in mouse and human models of ALS/FTD.

### Supplementary Information

ESM 1 (DOCX 10.8 MB)

## Data Availability

The datasets used and/or analysed during the current study are available from the corresponding author upon reasonable request.

## Abbreviations

ALS: Amyotrophic lateral sclerosis
EDL: Extensor digitorum longus
FTD: Frontotemporal dementia
FMRP: Fragile X mental retardation protein
FUS: Fused in Sarcoma
G3BP: Ras-GAP SH3 domain binding protein
GFAP: Glial fibrillary acid protein
HSF-1: Heat shock factor-1
HSP: Heat shock protein
HSP70: Heat shock protein 70
HSR: Heat shock response
IBMPFD: Inclusion body myopathy with Paget’s disease of bone and frontotemporal dementia
iPSC: Induced pluripotent stem cell
LC3: Microtubule-associated protein 1A/1B-light chain 3
MAPT: Microtubule-associated protein tau
MN: Motor neuron
MSP: Multisystem proteinopathy
mVCP: Mutant human valosin-containing protein
PD: Paget’s disease of bone
SBMA: Spinal and bulbar muscular atrophy
SOD: Superoxide dismutase
TDP-43: TAR DNA-binding protein 43
UPS: Ubiquitin-proteasome system
VCP: Valosin-containing protein
wtVCP: Wildtype human valosin-containing protein
WT: Wildtype
