# Supplementary material for: Amplifying the Heat Shock Response Ameliorates ALS and FTD Pathology in Mouse and Human Models
Source: Mol Neurobiol. 2023 Jul 29;60(12):6896–915. doi: 10.1007/s12035-023-03509-2 (PMC10657827; doi:10.1007/s12035-023-03509-2)
Supplement: Supplementary file 1 — (DOCX 10.8 MB) [file 12035_2023_3509_MOESM1_ESM.docx]

**Amplifying the heat shock response ameliorates ALS and FTD pathology in mouse and human models.**

Mhoriam Ahmed^1^, Charlotte Spicer^2^, Jasmine Harley^3^, J Paul Taylor^4^, Michael Hanna^5^, Rickie Patani^6^, Linda Greensmith^*^

*Correspondence to Professor Linda Greensmith, Department of Neuromuscular Diseases, UCL Queen Square Institute of Neurology, London WC1N 3BG, UK

Email: [l.greensmith@ucl.ac.uk](mailto:l.greensmith@ucl.ac.uk)

Telephone: 0203 448 4280

**Supplementary Materials and Methods**

**Immunohistochemistry**

For immunofluorescent labelling, frozen sections were blocked for 1 hour at room temperature in blocking solution (10% normal goat serum in PBS + 0.1% Triton X-100), followed by incubation with primary antibodies against phosphorylated tau (AT8 Pierce Endogen Mouse monoclonal 1:100), Iba1 (Wako Chemicals 019-19741 Rabbit polyclonal 1:100), GFAP-Cy3 (Sigma G-A-5 Mouse monoclonal 1:1000), β-III tubulin (TUJ1, Cambridge Bioscience 3525-100 Rabbit polyclonal 1:100), Tia1 (Abcam ab205063 Rabbit polyclonal 1:50), G3BP (BD Bioscience Mouse monoclonal 1:200), FMRP (Abcam 1D10 Mouse monoclonal 1:200), for 1 hour at room temperature. Sections were washed in PBS and incubated for 2 hours at room temperature with the appropriate fluorescently labelled secondary antibodies and 4’, 6-Diamidino-2-Phenylindole (DAPI; Sigma) to label nuclei. Sudan black was applied to sections for 10 minutes to quench autofluorescence prior to coverslip mounting. Tissue sections from three mice per experimental group were assessed for each antibody tested and compared to negative controls run simultaneously. Final fluorescent images were visualised under a Leica fluorescent microscope and analysed using Leica Application Suite software (Leica Microsystems, Germany).

**TUNEL Staining**

The TACS^®^ 2 TdT Fluorescein kit (Trevigen) was used for in situ detection of apoptosis by TUNEL in VCP patient fibroblasts using the manufacturer’s instructions. Coverslips were counterstained with DAPI to label nuclei. Images were visualised using a Leica fluorescent microscope.

**Supplementary Figures**


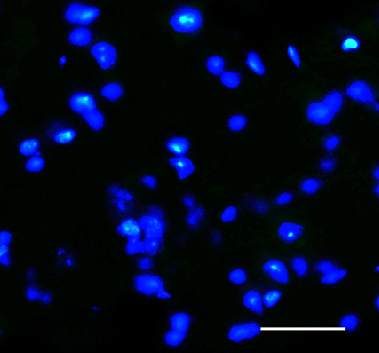

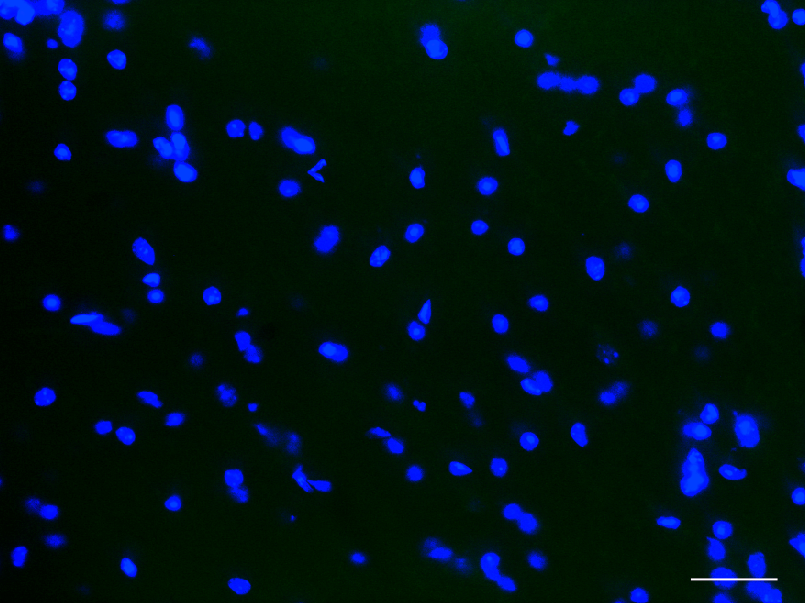

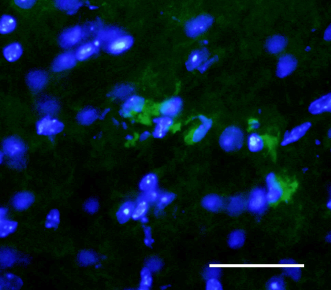

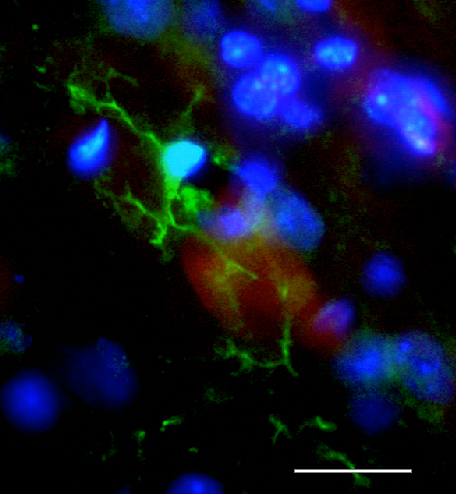

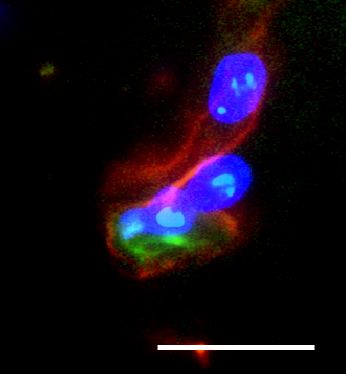

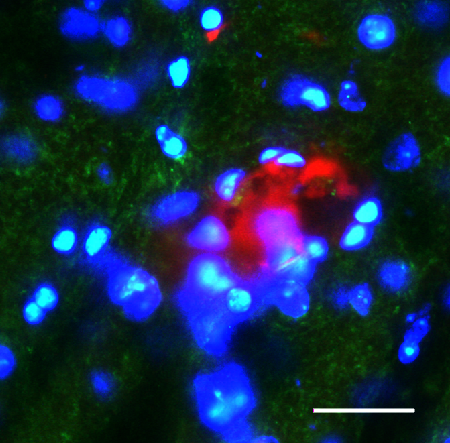


**Supplementary Fig 1**

p-tau

β-III tubulin

mVCP

mVCP

p-tau

Iba1

mVCP

10µm

10µm

10µm

**A**

mVCP + arimoclomol

mVCP + arimoclomol

mVCP + arimoclomol

GFAP

p-tau


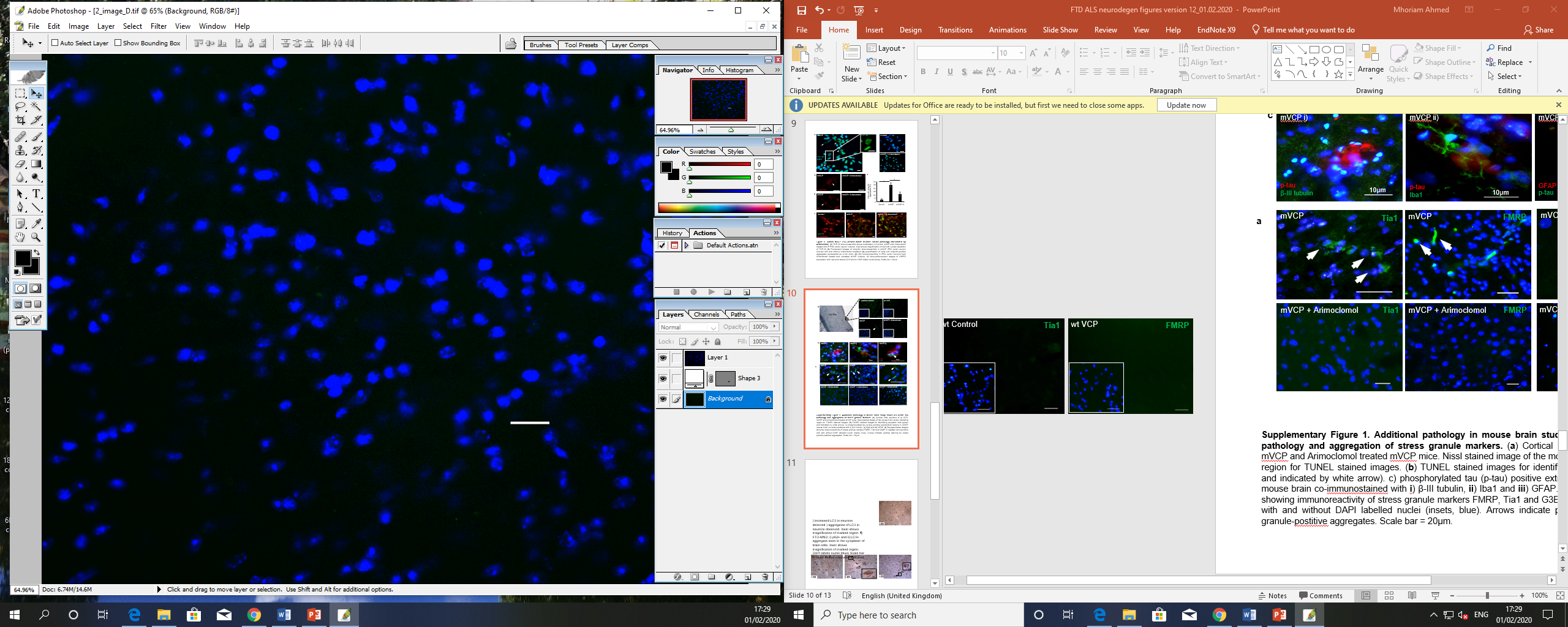


**Tia1**

**FMRP**

**G3BP**

wtVCP

wtVCP

wtVCP

**B**

**Tia1**


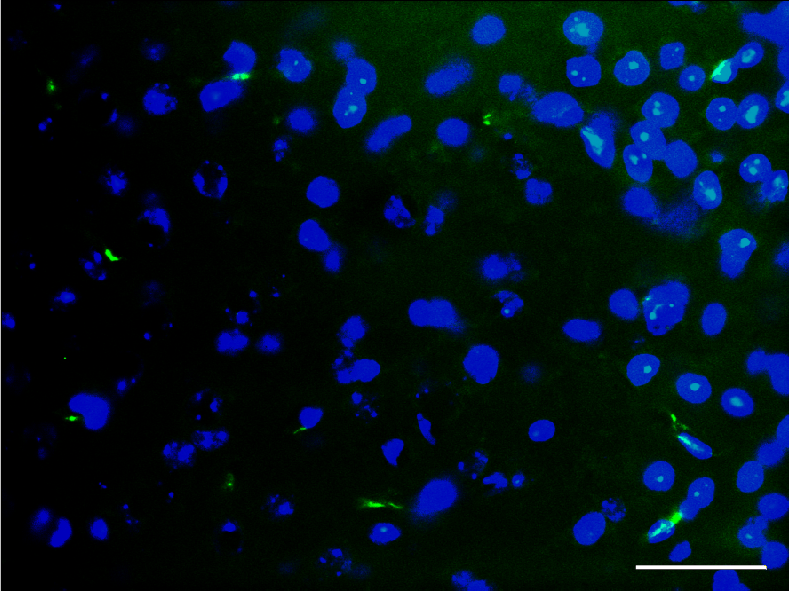


mVCP

**G3BP**


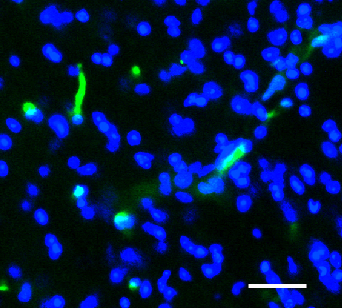


mVCP

**FMRP**

mVCP


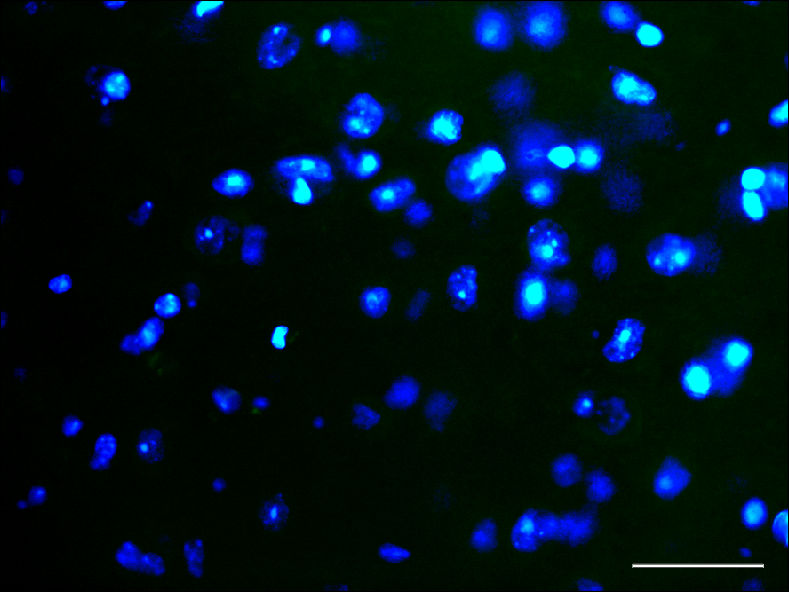

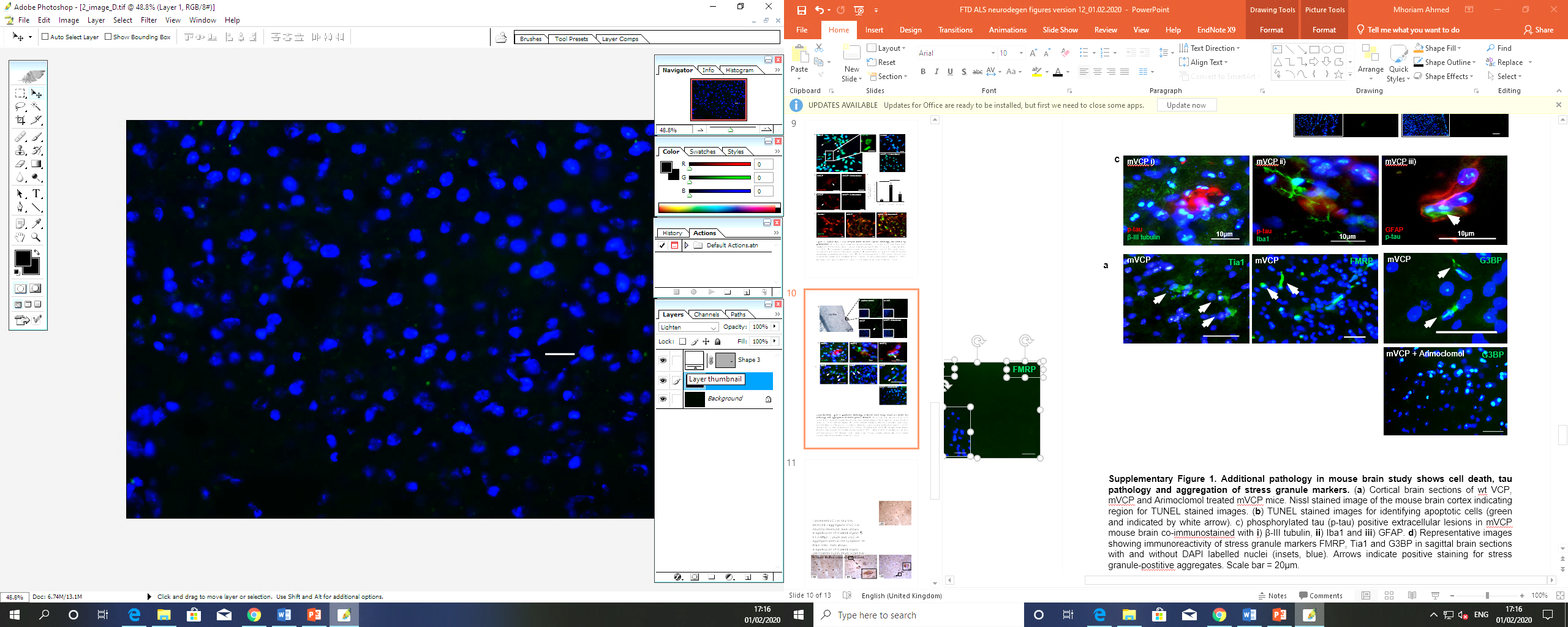

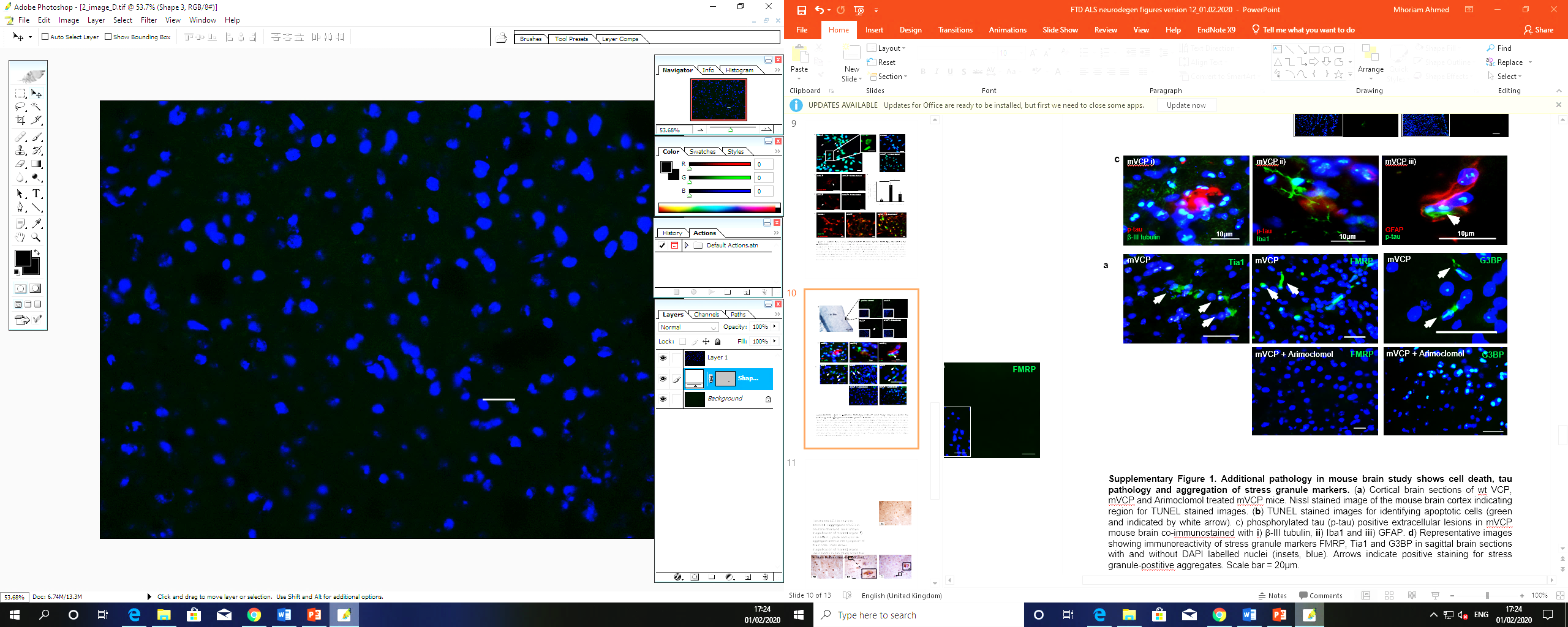


**Tia1**

**FMRP**

**G3BP**

**Sup Fig.1** **Additional pathology in mouse brain study shows tau pathology and aggregation of stress granule markers.** (**A**) Phosphorylated tau (p-tau) positive extracellular lesions in mVCP mouse brain co-immunostained with β-III tubulin, Iba1 and GFAP. (**B**) Representative images showing immunoreactivity of stress granule markers FMRP, Tia1 and G3BP in sagittal brain sections with and without arimoclomol treatment. DAPI labels nuclei (blue). Arrows indicate stress granule-positive aggregates. Scale bar = 20µm unless otherwise indicated.


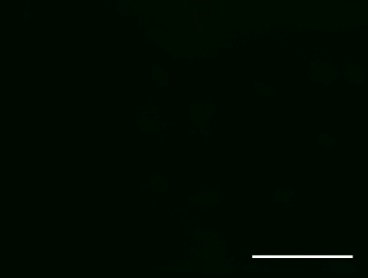

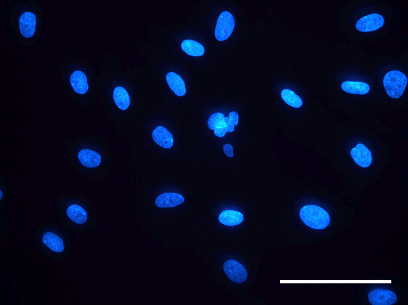

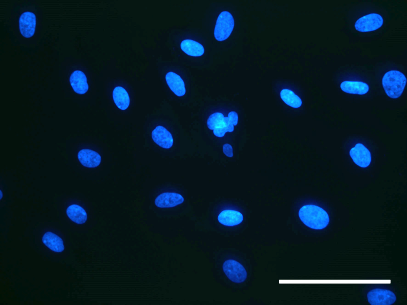

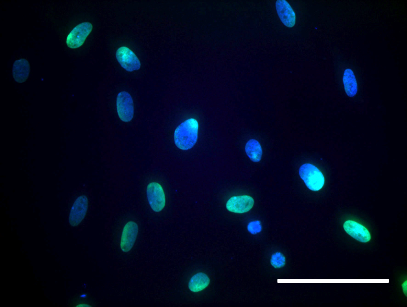

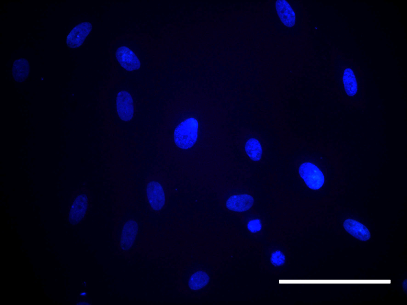

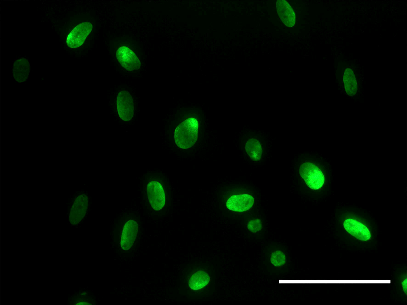


**Supplementary Fig 2.**

DAPI

Merge

TUNEL

Untreated mVCP

DAPI

Merge

TUNEL

Positive control

TUNEL

**B**

**A**


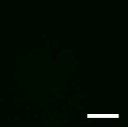

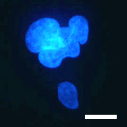

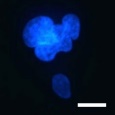


**Sup Fig 2. Abnormal nuclear morphology in mutant VCP patient fibroblasts show no evidence of apoptosis.**  TUNEL analysis of (**A**) untreated patient fibroblasts and (**B**) a TACS-Nuclease-treated positive control sample, co-stained with the nuclear marker DAPI (blue). Arrows indicate a disrupted nucleus that is TUNEL-negative. Scale bar= 50μm. Insets show higher magnification. Scale bar= 10μm.

**Supplementary Table 1**. **FTD patient information**

Details of age, sex, post-mortem (PM) delay and diagnosed sub-classification for patients and healthy control from whom cortical brain samples were obtained for this study. No further information regarding the genetic diagnosis or phenotype was available for these patients.

| **Patient no.** | **Patient code** | **Age** | **Sex** | **PM delay**  **(hours)** | **FTD sub-classification** |
| --- | --- | --- | --- | --- | --- |
| 1 | P6/07 | 66 | M | 80.45 | FTD associated with tau mutation |
| 2 | P65/10 | 62 | M | 93.00 | FTD associated with mutation in TDP-43 |
| 3 | P27/05 | 70 | M | 9.50 | FTD with ubiquitinated inclusion bodies |
| 4 | P28/07 | 74 | M | 19.00 | FTD associated with motor neuron disease |
|  | **Control code** |  |  |  |  |
|  | P44/02 | 78 | F | 23.30 |  |
|  | P47/11 | 79 | F | 78.50 |  |
|  | P94/05 | 71 | M | 38.50 |  |
|  |  |  |  |  |  |
|  |  |  |  |  |  |

DAPI

Merge

TUNEL

DAPI
